# Glibenclamide Attenuates Neuroinflammation and Promotes Neurological Recovery After Intracerebral Hemorrhage in Aged Rats

**DOI:** 10.3389/fnagi.2021.729652

**Published:** 2021-08-26

**Authors:** Bing Jiang, Ying Zhang, Yan Wang, Zheng Li, Qianwei Chen, Jun Tang, Gang Zhu

**Affiliations:** ^1^Department of Neurology, Chengdu Fifth People’s Hospital, Chengdu, China; ^2^Department of Neurosurgery, Southwest Hospital, Army Medical University, Chongqing, China

**Keywords:** intracerebral hemorrhage, glibenclamide, SUR1, neuroinflammation, aged rats

## Abstract

Intracerebral hemorrhage (ICH) is a common disease in the elderly population. Inflammation following ICH plays a detrimental role in secondary brain injury, which is associated with a poor prognosis of patients with ICH, and no efficient pharmacological preventions are available. Here, we investigated the effects of glibenclamide (GLC) on neuroinflammation in an autoblood-induced aged rat (18 months old) model of ICH. Rats were randomized into the sham, vehicle, and GLC groups. First, we investigated the expression level of sulfonylurea receptor 1 (Sur1) surrounding the hematoma after ICH. Then, neurological scores were calculated, and water maze tests, brain water content analysis, western blotting, and immunofluorescence assays were implemented to detect the neuroprotective effect of GLC. The expression of the Sur1-Trpm4 channel was significantly increased in the perihematomal tissue following ICH in aged rats. The GLC administration effectively reduced brain edema and improved neurofunction deficits following ICH. In addition, GLC increased the expression of brain-derived neurotrophic factors and decreased the expression of proinflammatory factors [tumor necrosis factor (TNF)-α,interleukin (IL)-1, and IL-6]. Moreover, GLC markedly reduced Ikappa-B (IκB) kinase (IKK) expression in microglia and nuclear factor (NF)-κB-P65 levels in perihematomal tissue. GLC ameliorated ICH-induced neuroinflammation and improved neurological outcomes in aged rats. In part, GLC may exert these effects by regulating the NF-κB signaling pathway through the Sur1-Trpm4 channel.

## Introduction

Intracerebral hemorrhage (ICH) is a subtype of stroke that leads to high rates of disability and death in humans (Xi et al., 2006). ICH is a common disease among the elderly population, and age is an essential factor that affects the prognosis of humans and animals after a stroke (Davis et al., 1995; Gong et al., 2004). Inflammatory responses play an important role in the pathogenesis after ICH (Aronowski and Hall, 2005; Hussain et al., 2009). Previous research has shown that nuclear factor (NF)-κB plays a crucial role in secondary brain injury after ICH (Pozniak et al., 2014; Zhang et al., 2019). Therefore, it is necessary to research new therapies that target inflammatory responses to improve the prognosis of patients with clinical ICH.

Glibenclamide (GLC) is an oral hypoglycemic medicine that works by inhibiting sulfonylurea receptor 1 (Sur1) (Kurland et al., 2013). Sur1 forms two distinct ion channels: the Sur1-Trpm4 channel and Sur1-Kir6.2 channel (Chen et al., 2003). Many reports have shown that GLC protects against central nervous system (CNS) diseases, such as cerebral metastases, subarachnoid hemorrhage (SAH), traumatic brain injury, ischemic stroke, and status epilepticus (Simard et al., 2009; Thompson et al., 2013; Lin et al., 2017; Jha et al., 2020; Woo et al., 2020). For the first time, our previous research showed that the expression increased in the Sur1-Trpm4 channel in the perihematomal tissue following ICH in adult rats. GLC treatment improved neurological outcomes and protected the blood–brain barrier integrity following ICH, and these effects involved the expression of MMPs (Jiang et al., 2016). Previous research demonstrated that GLC treatment reduced tumor necrosis factor (TNF)-α, interleukin (IL)-6, and NF-κB following experimental cardiac arrest (Nakayama et al., 2018). However, no literature reported whether CLC participates in neuroinflammation following ICH in aged rats.

Thus, we hypothesize that GLC treatment alleviates secondary brain injury and improves neurofunction deficits after ICH in aged rats by suppressing neuroinflammation by inhibiting the Sur1-Trpm4 channel. An ICH model of an aged rat was used to verify this hypothesis.

## Materials and Methods

### Ethics Statement

All the procedures in this study complied with the Guide for the Care and Use of Laboratory Animals. All the experiments were designed to minimize pain and animal numbers, and the study protocol was approved by the Animal Care and Use Committee at Army Medical University. The animals were housed under a 12-h light and 12-h dark cycle and were given free access to food and water.

### Animals and Surgical Procedures

A total of 150 male Sprague-Dawley (SD) rats, weighing 450–550 g, were provided by the Army Medical University (Chongqing, China). Rats were randomly divided into three groups: the sham-operated group, ICH + vehicle group, and ICH + GLC group. To mimic the clinical condition of ICH, a model was established *via* injection of autogenous blood, as previously reported (Jiang et al., 2016). Briefly, the animals were anesthetized by intraperitoneal injection of pentobarbital (40 mg/kg). The body temperature of the animals was maintained at 37°C. The animals were positioned in a stereotaxic frame, a cranial burr hole (1 mm diameter) was drilled, and 100 μl autogenous arterial blood (obtained from the right femoral artery) was microinfused using a pump at a constant rate of 10 μl/min into the right caudate nucleus (coordinates: 3.5 mm lateral, 5.5 mm ventral, and 0.2 mm anterior to the bregma) through a 29-G needle. The sham-operated rats were only subjected to needle insertion. All rats survived the ICH induction and no mortality happened.

### Glibenclamide Treatment

Glibenclamide (Tocris Bioscience, Ellisville, MO, United States) was administered as previously reported (Jiang et al., 2016). Briefly, dimethyl sulfoxide (DMSO) (50 mg/ml) was used to prepare stock solutions of GLC. The injection solution (200 ng/μl or 1 μg/ml) was made by dilution in 0.9% NaCl, and clarifying the solution using a few microliters of 0.1 N NaOH (final pH ∼ 8.5). At the end of the surgery, GLC was administered in a single loading dose (10 μg/kg intraperitoneal injection) plus continuous subcutaneous infusion (200 ng/h) *via* a mini-osmotic subcutaneous pump (Alzet, 2001, 1.0 μl/h; Alzet Corp., Cupertino, CA, United States). The vehicle group was treated with vehicle control solutions in the same manner.

### Measurement of Brain Water Content

The brain water content was examined 72 h following ICH, as previously reported (Li et al., 2015). Briefly, the rats (*n* = 10/group) were euthanized and decapitated, the brains were quickly removed, and a 4-mm thick section of the coronal brain tissue surrounding the needle entry site was harvested. The brain tissue was divided into four parts: the contralateral cortex, contralateral basal ganglia, ipsilateral cortex, and ipsilateral basal ganglia. The cerebellum served as the internal control. The brain tissue weights were determined immediately after removal and after drying for more than 24 h at 100°C on an electronic analytical balance. The brain water content (%) was calculated using the following formula: (wet weight−dry weight)/wet weight × 100%.

### Tissue Fixation and Immunofluorescence

Immunofluorescent labeling was conducted 24 or 72 h following ICH as previously described (Tang et al., 2015). About 18-μm thick brain tissue sections were prepared and stored at −20°C. The specimens were incubated with primary antibodies overnight at 4°C, and then with the appropriate secondary antibodies for 2 h at 37°C. Co-localization was examined by fluorescence microscopy (LSM780; Zeiss, Oberkochen, Germany).

### Western Blot Analysis

Western blot analysis was conducted 24 h following ICH as previously described (Qi et al., 2019). The perihematomal brain tissues (4-mm-thick) were sampled. The relative densities of the bands were analyzed using ImageJ software (National Institutes of Health, Bethesda, MD, United States).

### Real-Time PCR

PCR was performed to analyze Sur1 gene expression as previously reported (Jiang et al., 2016). Rats (*n* = 6/group) were sacrificed by decapitation 6, 12, and 24 h following ICH. The brain tissues were dissected (2 mm posterior and 2 mm anterior to the needle entry site) and immediately flash-frozen with liquid nitrogen. The primers for SUR1 were as follows: forward, 5′-CACAAGAAGCCCATCGACCT-3′; reverse, 5′-ATCGAAGGCCAAGCAGAGTC-3′ (Table 1). A positive standard curve was obtained using a serially diluted complementary DNA sample mixture. Gene expression was normalized by glyceraldehyde 3-phosphate dehydrogenase (GAPDH) expression and quantified with standard samples. The data are expressed as a normalized messenger RNA expression (fold mRNA increase).

**TABLE 1:** Primers used for real-time RT-PCR.

**Gene**	**GenBank accession no.**	**Primer sequences**
SUR1	NM_013039.2	F: CACAAGAAGCCCATCGACCT
		R: ATCGAAGGCCAAGCAGAGTC
GAPDH	NM_017008.4	F: TGAGGAGTCCCCATCCCAAC
		R: GATGGTATTCGAGAGAAGGGAGG
β-actin	NM_031144.3	F: GCAGGAGTACGATGAGTCCG
		R: ACGCAGCTCAGTAACAGTCC

*F, forward primer; R, reverse primer.*

### Corner Turn Test and Forelimb Placing Test

We used the corner turn test and forelimb placement test to assess the neurological function of the experimental rats 7 days following ICH as previously described (Hua et al., 2002; Krafft et al., 2014; Tan et al., 2017).

The corner turn test was conducted by two blinded observers. The experimental rats were allowed to proceed to a corner with an angle of 30°. We recorded the direction in which rats turned, and the process was repeated 10 times (60 s between trials); the percentage of right turns was calculated.

The forelimb placement test was conducted by two blinded observers. The rats were held by the torso, and all four limbs were allowed to hang freely in space. A trial was scored if a rat placed its forelimb on the edge of the countertop in response to vibrissae stimulation. Each forelimb was tested 10 times, and the percentage of successful scores was determined.

### Morris Water Maze Test

The Morris water maze test was performed to measure the learning and spatial memory of rats as previously described (Dai et al., 2017). Twenty-three days following ICH, rats (*n* = 10, per group) were placed in a pool (200 cm in diameter, 50 cm in depth) in which they searched to find a platform (5 cm in diameter, top surface 1.5 cm below the surface of the water) within 120 s. The rats that failed the mission would be picked up and placed on the platform for 15 s to familiar with the situation. The rats were subjected to five consecutive days of trials. The latency time was recorded to assess spatial learning ability. The probe trial was performed on the sixth day by removing the platform, and each rat was allowed to swim freely (120 s). The number of times the target area (previous location of the platform) was crossed, the percent distance and percent time in the target quadrant were analyzed.

### Statistical Analysis

The results of this study are expressed as the mean ± SD. Statistical analysis of the data was conducted using one-way analysis ANOVA, followed by Student–Newman–Keuls (SNK). Statistical significance was set as a *p*-value < 0.05.

## Results

### Sur1 Is Upregulated After ICH in Aged Rats

**FIGURE 1:**
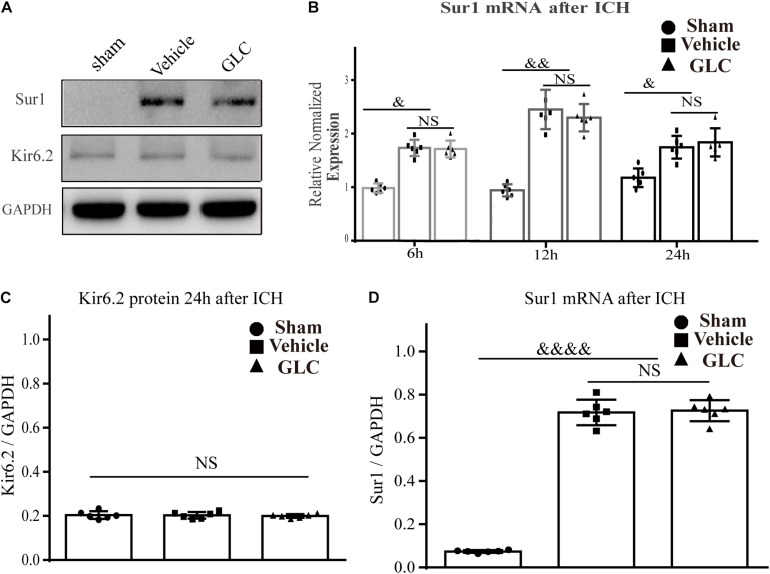
Sulfonylurea receptor 1 (Sur1), but not Kir6.2, is upregulated in the aged rat model of intracerebral hemorrhage (ICH). **(A,C,D)** Western blot analysis and quantification of the mean density of the Sur1 (180 kDa) and Kir6.2 (45 kDa) bands surrounding the hematoma 24 h after ICH (six rats/group). The results of the mean band densities are presented as the mean ± SD, ^&&&&^*p* < 0.0001. **(B)** Relative normalized expression levels of Sur1 mRNA surrounding the hematoma at 6, 12, and 24 h after ICH, Sur1 mRNA expression increased and peaked at 12 h (six rats/group). The results are presented as the mean ± SD, ^&^*p* < 0.05, ^&&^*p* < 0.01.

The protein expression of Sur1 was significantly upregulated surrounding the hematoma after ICH in aged rats (*p* < 0.0001, Figures 1A,D), but no significant difference was observed between the rats in the GLC treatment group and the vehicle group (*p* > 0.05, Figures 1A,D). No difference was observed in KIR6.2 expression among the sham group, GLC treatment group, and vehicle group (*p* > 0.05, Figure 1C). The Sur1 mRNA expression was examined at 6, 12, and 24 h after ICH. Compared with the sham-treated rats, aged rats with ICH exhibited a remarkable increase in Sur1 mRNA (*p* < 0.05, *p* < 0.01, Figure 1B).

### Glibenclamide Administration Improved Neurological Deficits

**FIGURE 2:**
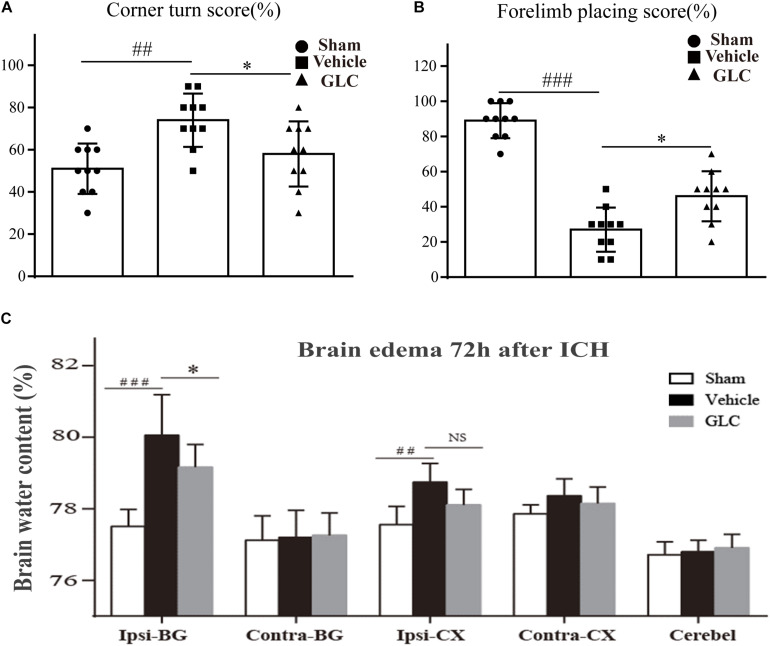
**(A)** Results of the corner turn test on day 3 after intracerebral hemorrhage (ICH). **(B)** Results of the forelimb placement test on day 3 after ICH thrombolytic therapy. The results are presented as the mean ± SD. ^∗^*p* < 0.05, ^##^*p* < 0.01, ^###^*p* < 0.001. **(C)** Glibenclamide (GLC) significantly reduced ICH-induced brain edema in the ipsilateral basal ganglia (Ipsi-BG) and ipsilateral cortex (Ipsi-CX) 72 h after injury. The cerebellum (Cerebel) served as the internal control. The values are expressed as the mean ± SD, *n* = 10. Ipsi-BG: vehicle vs. sham ^###^*p* < 0.001, vs. GLC ^∗^*p* < 0.05; Ipsi-CX: vehicle vs. sham ^##^*p* < 0.01, vs. GLC *p* > 0.05.

The corner turn score and forelimb placing score were used to measure the neurological function of the rats on day 7 after ICH. In these tests, the GLC-treated group had a lower corner turn score (*p* < 0.05, Figure 2A) and higher forelimb placing score (*p* < 0.05, Figure 2B) than the vehicle group.

### Treatment With GLC Decreased Brain Water Content

The brain water content of the rats in the vehicle group was significantly increased 72 h after ICH, especially in the ipsilateral basal ganglia (Ipsi-BG: sham vs. vehicle, *p* < 0.001, Figure 2C). The GLC treatment remarkably decreased the brain water content in the ipsilateral basal ganglia (Ipsi-BG: vehicle vs. GLC, *p* < 0.05, Figure 2C).

### Glibenclamide Treatment Ameliorated the Performance of the Experimental Rats in the Morris Water Maze Test

**FIGURE 3:**
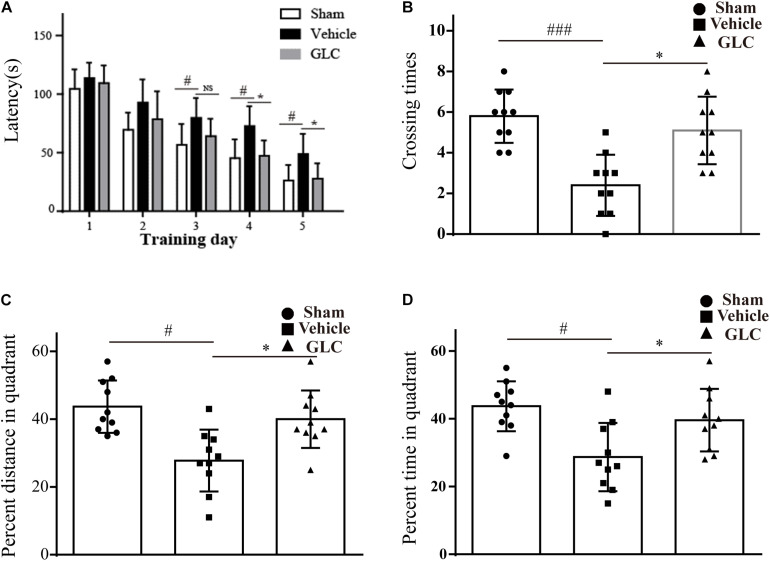
Spatial learning and memory deficits were examined 4 weeks after intracerebral hemorrhage (ICH). The values are expressed as the mean ± SD, *n* = 10. **(A)** Escape latency in training trials: vehicle vs. sham ^#^*p* < 0.05, vs. glibenclamide (GLC) ^∗^*p* < 0.05. **(B)** Times that the platform was crossed in the probe trials: vehicle vs. sham ^###^*p* < 0.001, vs. GLC ^∗^*p* < 0.05. **(C)** Percent distance in the target quadrant in the probe trials: vehicle vs. sham ^#^*p* < 0.05, vs. GLC ^∗^*p* < 0.05. **(D)** Percent time in the target quadrant in the probe trials: sham ^#^*p* < 0.05, vs. GLC ^∗^*p* < 0.05.

The rats exhibited disadvantageous spatial learning and memory deficits following ICH. The latency of the rats in the GLC group was significantly shorter than that of the rats in the vehicle group beginning on the fourth of five consecutive days of acquisition training (*p* < 0.05, Figure 3A). The GLC-treated rats spent more time (*p* < 0.05, Figure 3D) and traveled a greater distance (*p* < 0.05, Figure 3C) in the target quadrat compared with the vehicle-treated rats. In addition, the rats in the vehicle group crossed the platform fewer times (*p* < 0.001 and *p* < 0.05, respectively, vs. sham and GLC, Figure 3B). The results demonstrated that GLC improved spatial learning and memory deficits following ICH.

### Treatment With GLC Increased Brain-Derived Neurotropic Factor 72 h After ICH

**FIGURE 4:**
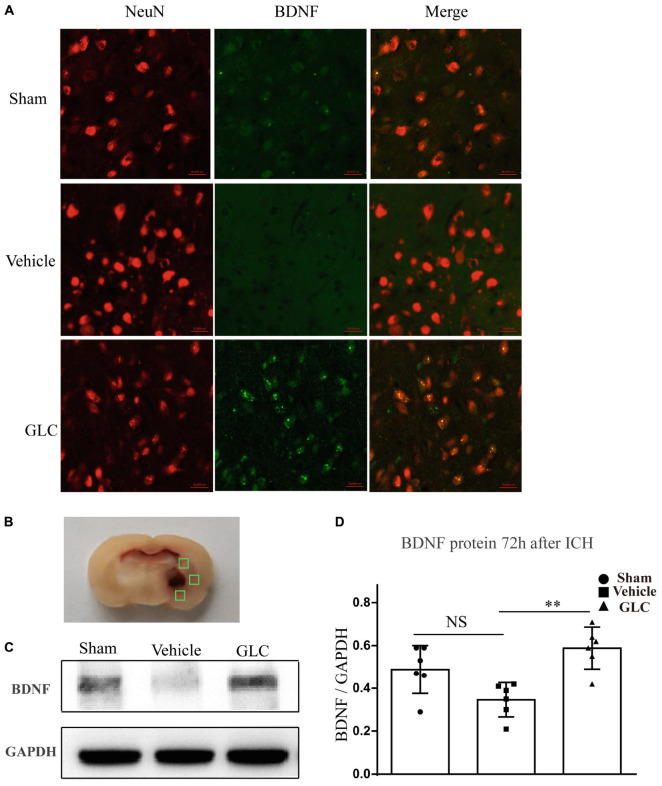
Glibenclamide treatment significantly increased the expression of brain-derived neurotrophic factor (BDNF). **(A)** BDNF upregulation was observed in neuron cells surrounding the hematoma (six rats/group). Bar = 20 μm. **(B)** Studied regions were marked with “□”. **(C,D)** The results of the mean band densities are presented as the mean ± SD, ^∗∗^*p* < 0.01.

Immunofluorescence staining suggested a significant increase in brain-derived neurotrophic factor (BDNF) co-localized with neurons in the GLC group compared with that in the vehicle group (Figure 4A). Studied regions were marked with “squares” (Figure 4B). Western blotting examination revealed that the GLC treatment significantly increased the protein expression of BDNF 72 h following ICH (*p* < 0.01, Figures 4C,D).

### Treatment With GLC Decreased the Expression of NF-κB

**FIGURE 5:**
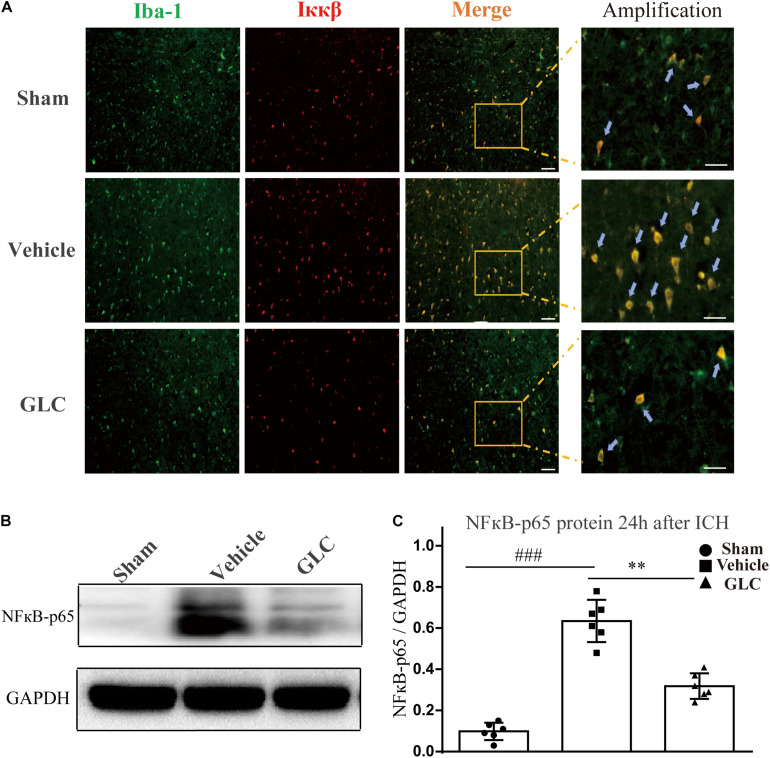
The regulatory effect of glibenclamide (GLC) on the NF-κB signaling pathway after intracerebral hemorrhage (ICH). **(A)** GLC reduced microglial secretion of Ikappa-B kinase (IKKβ) (*n* = 6). Bar = 20 μm. **(B,C)** Western blot analysis and quantification of the mean density of the nuclear factor (NF)-κB-p65 (65 kDa) band surrounding the hematoma 24 h after ICH (six rats/group), vehicle vs. sham ^###^*p* < 0.001, vs. GLC ^∗∗^*p* < 0.01. Amplified regions were marked with “squares”. Iba-1 co-localized with Ikkβ were marked with “arrows”.

We used western blotting and immunofluorescence staining to measure the expression of components of the NF-κB signaling pathway. Immunofluorescence staining demonstrated a remarkable decrease in Ikappa-B kinase (IKKβ) in the GLC group compared with the vehicle group (Figure 5A). The protein expression of NF-κB-p65 was significantly increased 24 h after ICH (sham vs. vehicle, *p* < 0.001, Figures 5B,C). The GLC treatment significantly decreased the protein expression of NF-κB p65 24 h after ICH (vehicle vs. GLC, *p* < 0.01, Figures 5B,C).

### Glibenclamide Reduces ICH-Induced Neuroinflammation

**FIGURE 6:**
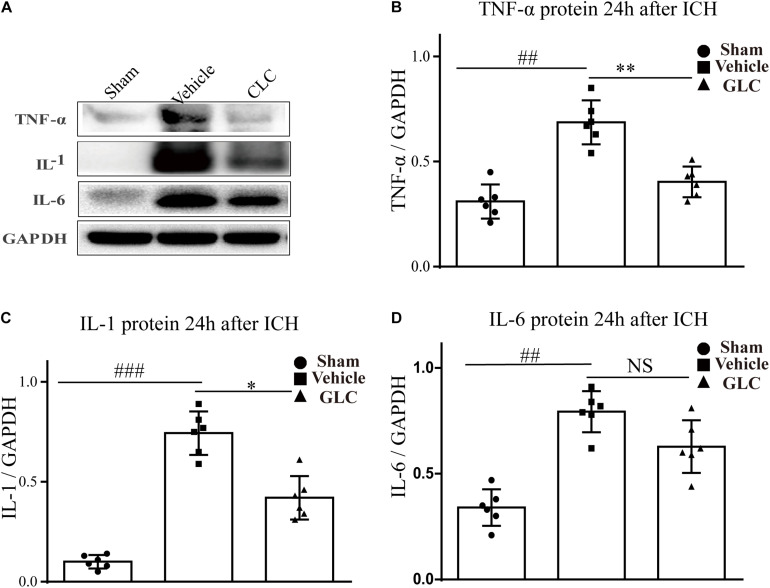
Effect of glibenclamide (GLC) on neuroinflammation protein expression following intracerebral hemorrhage (ICH). The band intensity quantification is presented as the mean ± SD, **(A,B)** tumor necrosis factor (TNF)-α: vehicle vs. sham ^##^*p* < 0.01, vs. GLC ^∗∗^*p* < 0.01; **(A,C)** IL-1: vehicle vs. sham ^###^*p* < 0.001, vs. GLC ^∗^*p* < 0.05; **(A,D)** IL-6: vehicle vs. sham ^##^*p* < 0.01, vs. GLC *p* > 0.05.

The expression of inflammatory factors was examined using western blotting analysis. The results revealed that TNF-α, IL-1, and IL-6 expression was significantly increased 24 h after ICH (Figure 6). GLC significantly decreased the expression of TNF-α (*p* < 0.01, Figure 6A,B). GLC significantly reduced the expression of IL-1 (*p* < 0.05, Figure 6A,C). GLC decreased the expression of IL-6, but no significant difference was observed (*p* > 0.05, Figures 6A,D).

## Discussion

Intracerebral hemorrhage is currently one of the most common diseases, particularly in the elderly population. With an aged rat model, we mimicked the pathophysiological processes observed in spontaneous ICH in elderly patients in the clinic. We detected an upregulation of Sur1 expression in an ICH model of an aged rat. Moreover, we suggested that the inhibition of Sur1 by GLC ameliorated neuroinflammation and improved neurological deficits.

Sulfonylurea receptor 1 forms two distinct ion channels: the Sur1-Trpm4 channel and Sur1-Kir6.2 channel (Tosun et al., 2013). Under normal conditions, Sur1 is constitutively expressed in some neurons of the CNS and exclusively forms Sur1-Kir6.2 channels (Simard et al., 2014). Previous study demonstrated, the expression increased in the Sur1-Trpm4 channel following ICH, which was not observed in uninjured brain tissues (Woo et al., 2013). We showed that the level of Sur1 was significantly increased 6 h following ICH in aged rats, and the increase continued until 12 h but decreased at 24 h. Interestingly, we found that the increased level and peak timepoint are somewhat inconsistent with the results of our previous study showing that Sur1 mRNA significantly increased 12 h after ICH in adult rats, and the increase was maintained until 24 h but decreased at 48 h. Several factors may explain this difference. One possible reason is related to the different measures of thrombin. Ibbotson et al. (1992) proved that coagulation rates in plasma are accelerated with age, suggesting that hematoma can produce much more thrombin in aged rats. Thrombin can induce the expression of matrix metalloproteinase (MMP)-9, and MMPs are involved in the expression of Sur1 (Caffes et al., 2015). The second possible explanation for our findings is related to Sur1 expression in the different microglial responses in adult and aged animals (Camacho et al., 2015). Microglia play an important role in inflammation after CNS injury, and inflammation may be a factor in the upregulation of Sur1 (Simard et al., 2009). Wasserman et al. (2008) reported a difference in microglial activation and macrophage distribution between young and aged rats following ICH. Therefore, it is possible that the timing of Sur1 upregulation after ICH might be different between young rats and aged rats. In this report, we demonstrate that the expression of Sur1 is upregulated in aged rats after ICH.

The present study shows that the Sur1-Trpm4 channel is implicated in ICH-induced inflammation. GLC administration significantly reduces the expression of the NF-κB, IL-1, TNF-α, and IL-6. Similarly, many studies have shown that GLC inhibits inflammation in animal models of SAH (Simard et al., 2009), experimental cardiac arrest (Nakayama et al., 2018), and cerebral ischemic injury (Caffes et al., 2015). Our previous study determined that inhibition of Sur1 alleviated ICH-induced metalloproteinase (MMPs) expression. Many previous studies showed that MMPs play an important role in neuroinflammation (Vandooren et al., 2014; Hannocks et al., 2017; Mi et al., 2021). The increased activity of MMPs can affect the secretion of many types of inflammatory cytokines and the activation of inflammatory cells (Florczak-Rzepka et al., 2012).

This study found that treatment with GLC induced the expression of BDNF. Previous studies determined that BDNF stimulates neural progenitor cells to differentiate into mature neurons, and it exerts a neurotrophic effect at sites of injury (Shimotake et al., 2010; Deng et al., 2019). Previous studies have shown that neuroinflammation reduces the expression of BDNF and negatively affects many stages of neurogenesis (Hashimoto, 2015; Zhang et al., 2018; Zhong et al., 2020). Bi et al. (2016) proved that neuroinflammation attenuates the expression of BDNF by activating the NF-κB pathway. Therefore, a significant increase in BDNF may be involved in the activation of the NF-κB pathway.

Many factors are related to neurological deficits following ICH, including primary brain injury, edema, inflammation, and age (Keep et al., 2012; Guo et al., 2021). In this study, GLC improved neurological deficits, consistent with previous studies (Liew et al., 2012; Xu et al., 2015). Simard et al. (2006) proved that continuous subcutaneous infusion of GLC (75 ng/h) reached the peri-infarct regions of rats with cerebral ischemia, resulting in potential neuroprotection. Several recent clinical trials have shown that GLC is associated with improvements in midline shift, level of alertness, neurofunction deficits, and survival after large hemispheric infarction (Kimberly et al., 2018; Sheth et al., 2018; Vorasayan et al., 2019). Robert et al. (2020) concluded that inhibiting the ion channel Sur1-Trpm4 could be a valuable adjuvant to prevent and even reverse fluid accumulation in the brain parenchyma. Several clinical trials about the safety and efficacy of GLC in CNS diseases are ongoing, such as SE-GRACE and GASH. Within a few years, all these research findings make it possible to use GLC in clinical stroke treatment.

## Conclusion

In summary, in the current study, we found that the expression of the Sur1-Trpm4 channel was significantly increased in perihematomal tissue following ICH in aged rats. We demonstrated that GLC ameliorated ICH-induced neuroinflammation and improved neurological outcomes, and GLC may exert these effects in part by regulating the NF-κB signaling pathway through the Sur1-Trpm4 channel. Our findings may contribute to the further elucidation of the mechanism of action of CLC and assist in developing a novel therapeutic strategy for treating clinical stroke.

## Data Availability Statement

The raw data supporting the conclusions of this article will be made available by the authors, without undue reservation.

## Ethics Statement

The animal study was reviewed and approved by the Animal Care and Use Committee at Army Medical University.

## Author Contributions

BJ and YZ wrote the manuscript, analyzed the data, and designed and performed the experiments. YW, ZL, JT, and QC assisted with the experiments, prepared the figures, and performed the behavioral tests. GZ contributed to the conception of the review and gave final approval of the version to be published. All authors contributed to the article and approved the submitted version.

## Conflict of Interest

The authors declare that the research was conducted in the absence of any commercial or financial relationships that could be construed as a potential conflict of interest.

## Correction Note

A correction has been made to this article. Details can be found at: 10.3389/fnagi.2026.1809579.

## Publisher’s Note

All claims expressed in this article are solely those of the authors and do not necessarily represent those of their affiliated organizations, or those of the publisher, the editors and the reviewers. Any product that may be evaluated in this article, or claim that may be made by its manufacturer, is not guaranteed or endorsed by the publisher.
