# Correction: Glibenclamide attenuates neuroinflammation and promotes neurological recovery after intracerebral hemorrhage in aged rats

**DOI:** 10.3389/fnagi.2026.1809579

**Published:** 2026-03-17

**Authors:** Bing Jiang, Ying Zhang, Yan Wang, Zheng Li, Qianwei Chen, Jun Tang, Gang Zhu

**Affiliations:** 1 Department of Neurology, Chengdu Fifth People's Hospital, Chengdu, China; 2 Department of Neurosurgery, Southwest Hospital, Army Medical University, Chongqing, China

**Keywords:** intracerebral hemorrhage, glibenclamide, SUR1, neuroinflammation, aged rats

There was a mistake in Figure 4A as published. There was an error in the sham group figure content. The corrected Figure 4A appears below.

**Figure 4:**
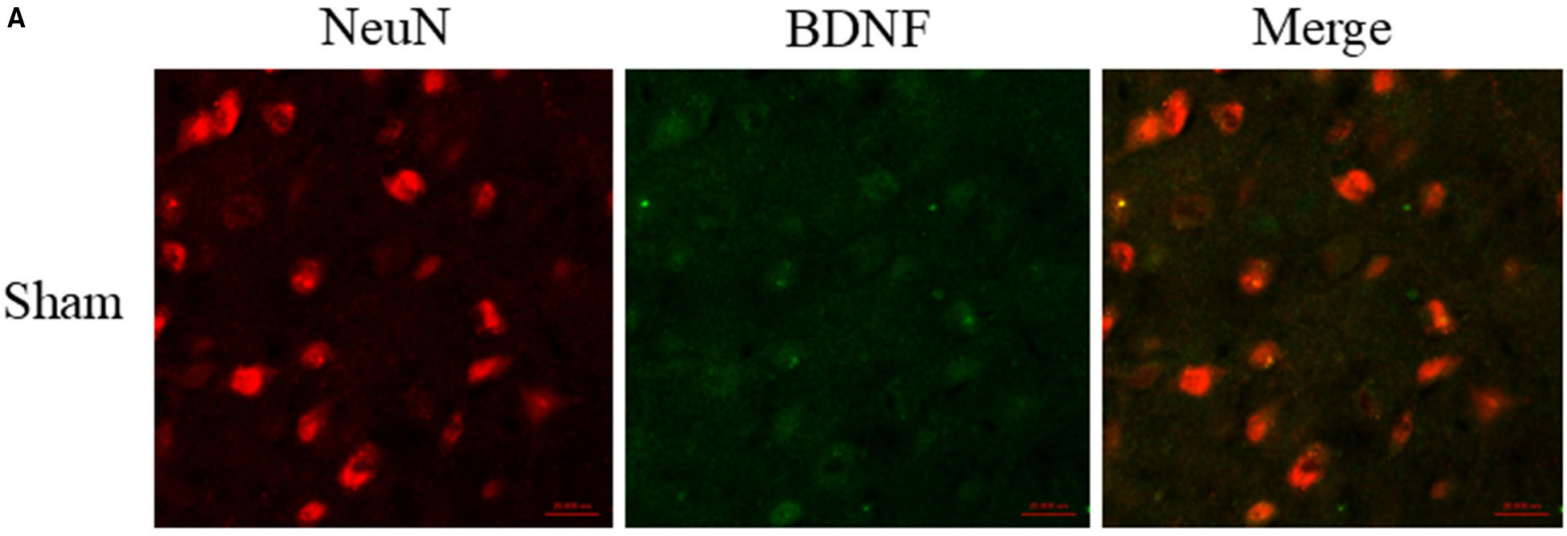
Glibenclamide treatment significantly increased the expression of brain-derived neurotrophic factor (BDNF). **(A)** BDNF upregulation was observed in neuron cells surrounding the hematoma (six rats/group). Bar = 20 μm. **(B)** Studied regions were marked with “□”. **(C, D)** The results of the mean band densities are presented as the mean ± SD, ^**^p < 0.01.

The original version of this article has been updated.

